# Identification of a Novel Missense Mutation of *POLR3A* Gene in a Cohort of Sicilian Patients with Leukodystrophy

**DOI:** 10.3390/biomedicines10092276

**Published:** 2022-09-14

**Authors:** Antonino Musumeci, Francesco Calì, Carmela Scuderi, Mirella Vinci, Girolamo Aurelio Vitello, Sebastiano Antonino Musumeci, Valeria Chiavetta, Concetta Federico, Greta Amore, Salvatore Saccone, Gabriella Di Rosa, Antonio Gennaro Nicotera

**Affiliations:** 1 Oasi Research Institute—IRCCS, Via Conte Ruggero 73, 94018 Troina, Italy; 2 Department Biological, Geological and Environmental Sciences, University of Catania, Via Androne 81, 95124 Catania, Italy; 3 Department of Human Pathology of the Adult and Developmental Age, “Gaetano Barresi” University of Messina, Via Consolare Valeria 1, 98125 Messina, Italy

**Keywords:** hypomyelination, leukodystrophy, missense mutation, neurodegenerative disorder, POLR3A

## Abstract

Recessive mutations in the *POLR3A* gene cause POLR3-HLD (the second-most-common form of childhood-onset hypomyelinating leukodystrophy), a neurodegenerative disorder featuring deficient cerebral myelin formation. To date, more than 140 *POLR3A* (NM_007055.3) missense mutations are related to the pathogenesis of POLR3-related leukodystrophy and spastic ataxia. Herein, in a cohort of five families from Sicily (Italy), we detected two cases of patients affected by POLR3-related leukodystrophy, one due to a compound heterozygous mutation in the *POLR3A* gene, including a previously undescribed missense mutation (c.328A > G (p.Lys110Glu)). Our study used an in-house NGS gene panel comprising 41 known leukodystrophy genes. Successively, we used a predictive test supporting the missense mutation as causative of disease, thus this mutation can be considered “Likely Pathogenic” and could be as a new pathogenetic mutation of the *POLR3A* gene causing a severe form of POLR3-HLD.

## 1. Introduction

The terms POLR3-related hypomyelinating leukodystrophy (POLR3-HLD) and 4H leukodystrophy (or 4H) have been recently adopted to indicate overlapping clinical phenotypes featuring hypomyelination, hypodontia, hypogonadotropic, and hypogonadism (4H syndrome); ataxia, delayed dentition, and hypomyelination (ADDH); tremor-ataxia with central hypomyelination (TACH); leukodystrophy with oligodontia (LO); and hypomyelination with cerebellar atrophy and hypoplasia of the corpus callosum (HCAHC) [1]. *POLR3A*, *POLR3B* and *POLR1C* encode subunits of RNA polymerases III (Pol III). POLR3A and POLR3B are the two largest subunits of Pol III and form the active center of the enzyme. POLR1C is a subunit common to RNA polymerases I (Pol I) and III (Pol III) and it has been hypothesized to act as a scaffold for the assembly of the enzyme core [1,2]. Mutations in *POLR3A*, *POLR3B*, or *POLR1C* genes are associated with POLR3-HLD, inherited in an autosomal recessive manner [3]. The dysregulation of transfer RNA (tRNA) expression leads to perturbed cytoplasmic protein synthesis in the brain [1] and impairs the function of Pol III-transcribed non-coding RNAs (ncRNAs), that play a pivotal role in development and maintenance of myelin [4,5].

**Figure 1:**
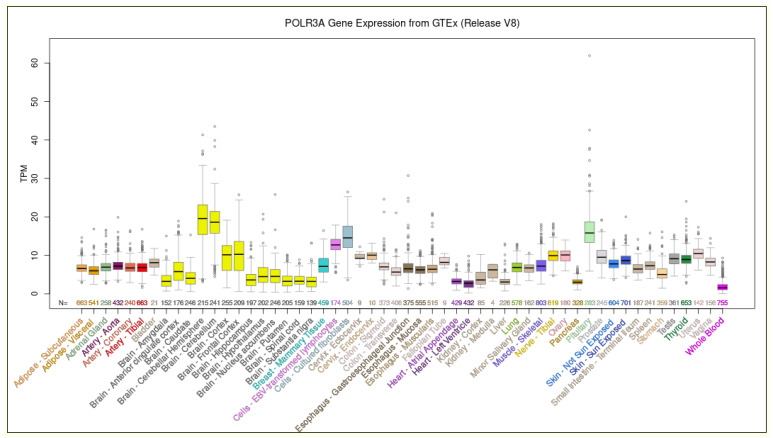
Expression levels of the *POLR3A* gene in human healthy tissues. The expression level was obtained in 54 human tissues from GTEx RNA-seq of 17,382 samples from 948 donors (V8, August 2019). TPM: transcripts per million. Data and image from the UCSC Genome Browser (http://genome.ucsc.edu, accessed on 23 August 2022).

The *POLR3A* gene spans a genomic region of 54,367 bp in the q22.3 band of human chromosome 10 and is composed by 31 exons. The coding sequence is 4170 nt, and the corresponding protein is composed by 1390 amino acids. *POLR3A* gene is constitutively expressed in all tissues. Although its expression does not reach very high levels, the highest level of expression was observed in the cerebellum (Figure 1). More than 140 POLR3A (NM_007055.3) missense mutations (Supplementary Table S1) are related to the pathogenesis of POLR3-related leukodystrophy and spastic ataxia [6].

In this study, we analyzed five Sicilian (Italian) patients with leukodystrophy, belonging to different families not related one to each other, using an in-house NGS panel. Two of these patients showed heterozygosity variants in the *POLR3A* gene, already described in the literature as causing leukodystrophy. One of them carried a single heterozygous mutation, and the other carried two heterozygous mutations; one of these mutations was located is in the 4th exon and the second in the 14th exon, two exon regions of this gene where several variants have previously been described. The mutation in exon 4 is a novel missense mutation ((c.328A > G) (p.Lys110Glu)), that we show here, for the first time, in a patient with a phenotype compatible with a severe form of POLR3- related leukodystrophy.

## 2. Materials and Methods

### 2.1. DNA Preparation

Genomic DNA was isolated from lymphocytes using the salt–phenol–chloroform extraction method, checked for degradation on agarose gel, and quantified by the Qubit 2.0 Fluorometer (Thermo Fisher Scientific, Foster City, CA, USA).

### 2.2. Genotypic Identification by NGS Sequencing

We used an in-house next-generation sequencing (NGS) gene panel comprising 41 known leukodystrophy genes (ABCD1, ARSA, ASPA, CSF1R, DARS2, EIF2B1, EIF2B2, EIF2B3, EIF2B4, EIF2B5, FAM126A, GALC, GFAP, GJC2, HSPD1, MCT8, MLC1, NOTCH3, PEX1, PEX10, PEX12, PEX13, PEX14, PEX2, PEX26, PEX3, PEX5, PEX6, PEX7, PLP1, POLR3A, PSAP, RANBP2, RNASEH2A, RNASEH2B, RNASEH2C, SAMHD1, SDHA, SOX10, SUMF1, TREX1).

The NGS panel was designed using the AmpliSeq Designer software (Life Technologies, CA, USA), targeting the complete coding sequence of the genes. The design target coverage was 98%. The amplicon library was prepared using the Ion AmpliSeq Library Kit 2.0 (Thermo Fisher Scientific, Foster City, CA, USA), then pooled together in equimolar concentrations using the Ion Library Equalizer Kit (Thermo Fisher Scientific, Foster City, CA, USA). Emulsion PCR, and ion sphere particle enrichment were carried out in the Ion One Touch 2 system, then loaded into an Ion 314 sequencing chip. Sequencing runs were performed using the Ion PGM 200 Sequencing kit. Data of runs were processed as previously described [7].

We filtered the identified variants according to: (i) recessive/de novo/X-linked pattern of inheritance, (ii) allele frequencies (mean average frequency, MAF) < 1% using as reference the following genomic datasets: 1000 Genomes, ESP6500, ExAC, and gnomAD. In silico analyses were performed with Varsome (ACMG criteria) [8,9] and by data obtained by ION reporter and wANNOVAR.

### 2.3. Sanger Sequencing

To confirm mutations identified in patients, Sanger sequencing was performed using the BigDye Terminator v1.1 Cycle Sequencing Kit (Thermo Fisher Scientific, Foster City, CA, USA), with an ABI 3130 Genetic Analyzer instrument (Thermo Fisher Scientific, Foster City, CA, USA).

## 3. Results

### 3.1. Study Subjects

**Figure 2:**
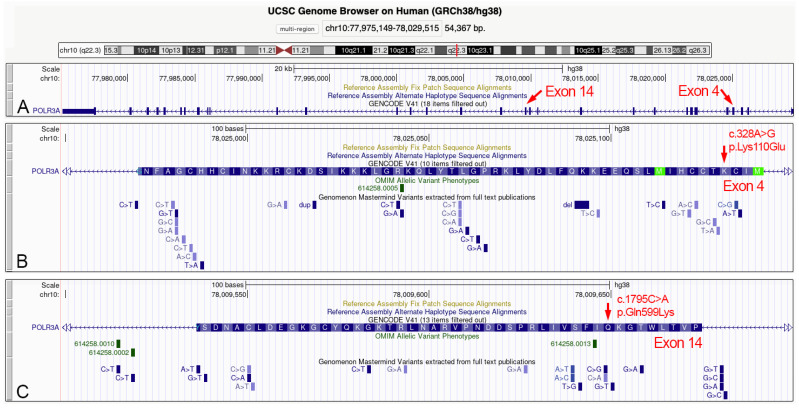
Localization of the two heterozygous mutations here described in the 5078-NP1688 patient. (**A**) Genomic organization of the *POLR3A* gene localized in the chromosomal band 10q22.3. Gene is transcribed form telomere to centromere side and consists of 31 exons. The exons 4 and 14 are highlighted and enlarged in the bottom panels. (**B**) Aminoacidic sequence codified by the exon 4 sequence. The 4th aminoacidic residue (lysine) corresponds to the mutated amino acid here identified for the first time. The currently described nucleotide mutations for the exon 4 sequence are shown at the bottom of panel B. (**C**) Aminoacidic sequence codified by the exon 14 sequence. The 9th aminoacidic residue (glutamine) corresponds to the mutated amino acid here observed in a POLR3-HLD patient, and previously described in the scientific literature. The currently described nucleotide mutations for the exon 14 sequence are shown at the bottom of panel C. Data and images from the UCSC Genome Browser (http://genome.ucsc.edu, accessed on 23 August 2022).

In this study, in-house NGS gene panel was tested in five families with a child in each of them affected by a severe form of hypomyelination leukodystrophy. The ethnicity of all participants was Caucasian (Sicily, Italy). Each family consisted of both parents with a child affected by leukodystrophy. Each trio was analyzed by NGS, and in two of these patients we detected causative mutations of the leukodystrophy (Table 1). In one of these patients, we identified variants in compound heterozygosity in the *POLR3A* gene already described in the literature, as causing leukodystrophy (Table 2). In a second patient, further to other polymorphisms in genes detected by our in-house NGS panel (Supplementary Table S2), we identified two *POLR3A* variants in compound heterozygosity. One mutation in the 4th exon and the other in the 14th exon, two regions of this gene where several variants have been previously described (Table 2, Figure 2, Supplementary Table S1).

The mutation in exon 4 is a novel missense mutation ((c.328A > G) (p.Lys110Glu)), that we show here for the first time, in a patient with a phenotype compatible with a severe form of POLR3-related leukodystrophy (see next paragraph).

### 3.2. Phenotypic Features of the Analyzed Subject

Here, we describe only the patient (5078-NP1688) in whom a novel mutation (p.Lys110Glu) has been identified. This patient is an Italian 20-year-old boy born to non-consanguineous parents after full-term pregnancy complicated by abortion risk. The proband presented a delayed psychomotor development (walking at 18 months with altered gait) and absence of language. Moreover, the patient showed difficulties obtaining food, growth failure, cataract, poor interaction abilities, and gradual worsening of neuromotor skills. His medical history was also positive for respiratory infections and constipation. The neurological examination revealed microcephaly, hyperreflexia, spastic diplegia, and axial hypotonia. At the age of 3 years, brain magnetic resonance imaging (MRI) showed slight hyperintensity, peri- and supraventricular white matter, and cortical atrophy.

**Figure 3:**
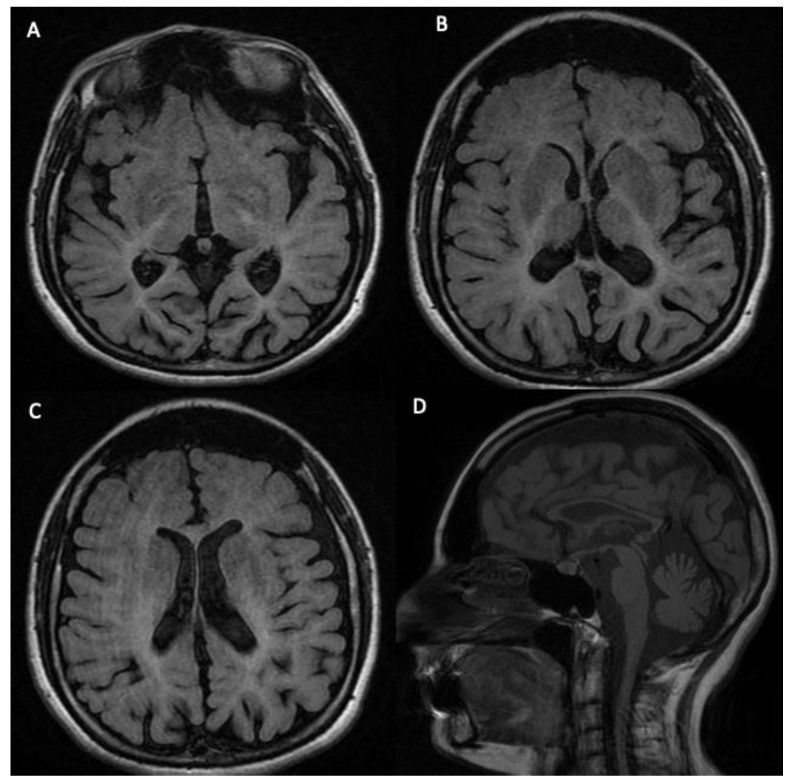
The brain MRI sequences show diffuse, bilateral, and relatively symmetrical cerebral white-matter hyperintensities, consistent with areas of hypomyelination, particularly evident in the subcortical and periventricular areas in T2-weighted and FLAIR images (**A**–**C**). In the same sequences, the extra-axial cerebrospinal fluid (CSF) spaces (including sulci, fissures, and ventricles), especially along the high-convexity area, are dilated. Sagittal T1-weighted image (**D**) shows a marked thinning of the corpus callosum, a verticalization of the tentorium, and olives and pontocerebellar hypoplasia. No signal/morphology alterations are evident in the cervical medulla.

Karyotype (46,XY), visual evoked potentials (VEPs), and metabolic blood and urine screening (including arylsulfatase and urinary organic acids) were within the normal range. The screening of the *PLP1* gene was also negative. At the age of 6 years, the patient presented nystagmus and severe worsening of gait abilities. A new VEP was performed, showing retrochiasmatic damage. The screening of the *GJC2* gene was negative. At the age of 8 years, a new brain MRI showed diffuse, bilateral, and relatively symmetrical cerebral white-matter hyperintensities, enlargement of the extra-axial cerebrospinal fluid (CSF) spaces, and in the sagittal T1-weighted image, a marked thinning of the corpus callosum, a verticalization of the tentorium, and olives and pontocerebellar hypoplasia (Figure 3). The EEG during sleep showed frontotemporal spike and sharp-wave.

At the age of 17 years, the patient was admitted to our hospital. The patient presented severe growth failure and microcephaly. Dental examination detected decalcification of teeth. Neurological examination showed tetraparesis with more severe hypertonia to upper limbs, hyperreflexia, inability to walk, Babinski sign and striatal toe. Eye examination revealed optic nerve atrophy, ptosis, astigmatism, and nystagmus. EEG, electrocardiogram, abdominal ultrasound, and metabolic blood and urine screening were not informative. Electromyography (EMG) showed myopathic findings.

### 3.3. Identification of the Missense Mutation in the POLR3A (NM_007055.3) Gene

**Figure 4:**
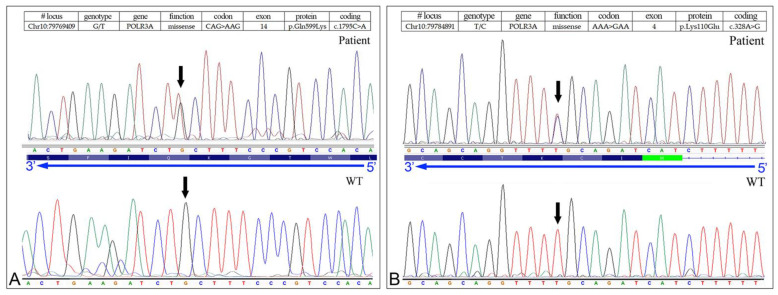
Sanger sequencing electropherograms showing, in the upper part, the compound heterozygous missense variants in the 5078-NP1688 patient. (**A**) c.1795C > A, (p.Gln599Lys) in the *POLR3A* gene (NM_007055.3). (**B**) c.328A > G (p.Lys110Glu) in the *POLR3A* gene (NM_007055.3). Bottom part: electropherograms of controls without mutations.

NGS analysis revealed a compound heterozygous missense mutation c.1795C > A, (p.Gln599Lys) paternally inherited (Figure 4, panel A), and c.328A > G (p.Lys110Glu), maternally inherited (Figure 4, panel B).

Although the missense mutation c.C1795A (p.Gln599Lys) was detected in other patients [10] and also described in subjects with POLR3-HLD, the other missense mutation c.328A > G (p.Lys110Glu) was not found in The Human Gene Mutation Database—Professional 2018.3 (HGMD) or in healthy controls (1000 Genomes Project, NHLBI-ESP6500 exome project). In addition, a predictive test performed with wANNOVAR (http://wannovar.wglab.org, accessed on 20 August 2022) confirmed that the missense mutation was causative of the disease (Table 3).

## 4. Discussion

The advent of NGS has made it possible to replace the “gene-by-gene” sequencing approach with a “panel of genes” strategy, benefiting mainly the diagnosis of complex genetic diseases such as leukoencephalopathies with high genetic heterogeneity (different genes responsible for the same clinical phenotype, and several mutations on each gene).

Recessive mutations in the *POLR3A* gene cause POLR3-HLD (the second-most-common form of childhood-onset hypomyelinating leukodystrophy—MIM #607694, #614381 #616494), a type of neurodegenerative disorder featured by deficient cerebral myelin formation [2]. Although *POLR3B* and *POLR1C* gene mutations are also associated with POLR3-HLD [3], several studies showed that *POLR3A* mutations lead to a more severe phenotype correlating with the age of onset disease [12].

**Figure 5:**
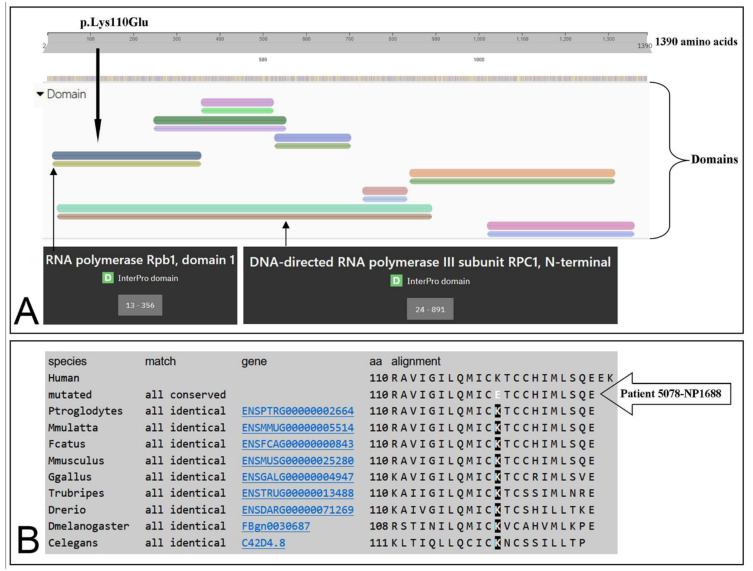
p.Lys110Glu domain position of POLR3A protein and sequence alignment. (**A**) Location of the p.Lys110Glu (black arrow) within RPC1 domain of the POLR3A protein (modified from InterPRO: https://www.ebi.ac.uk/interpro/protein/UniProt/O14802/, accessed on 6 September 2022). (**B**) Sequence alignment among multiple species showing that lysine (K) at position 110 is highly conserved, and mutated in the patient with leukodystrophy here analyzed (5078-NP1688) (modified from MutationTaster: www.mutationtaster.org/MT69/MutationTaster69.cgi, accessed on 6 September 2022).

In this study, involving some families from Sicily (Italy) we identified a compound heterozygous mutation, including an undescribed missense mutation in the *POLR3A* gene in a 20-year-old Italian male showing POLR3-HLD. In particular, the missense mutation c.328A > G (p.Lys110Glu) has not been previously described, and it should be stressed that this variant is placed in an evolutionarily conserved nucleotide site, as indicated by a Genomic Evolutionary Rate Profiling (GERP) value of 5.67. Indeed, the lysine at position 110 is highly conserved during evolution, being detected the same aminoacidic residue in nematodes (*C. elegans*), insects (*D. melanogaster*), and vertebrates, including *H. sapiens* (Figure 5). According to the American College of Medical Genetics (ACMG) [8,9] criteria, this missense mutation can be considered “Likely Pathogenic” and could be proposed to the international literature as a new pathogenetic mutation of *POLR3A* causing a severe form of POLR3-HLD.

## 5. Conclusions

NGS represents an innovation for screening molecular alterations in complex diseases. The use of NGS platforms has demonstrated its accuracy with a significant reduction in DNA sequencing costs compared with traditional testing. In particular, our panel was able to identify causative genetic variants of POLR3-related leukodystrophy in two out of five patients. Furthermore, these data could suggest a susceptibility of the Sicilian population to present mutations on this gene. However, this data should be further investigated and confirmed with further studies.

The present finding further expands our knowledge of pathogenic mutations of *POLR3A* gene. Indeed, although there is not currently a specific treatment to limit the clinical manifestations of POLR3-related leukodystrophy, an early diagnosis is useful to provide genetic counselling to family members to assess carrier status and establish genetic risk.

## Figures and Tables

**Table 1:** Patients with leukodystrophy analyzed with in-house NGS genes panel.

Sample	Age	Gender	Brain Magnetic Resonance Imaging (MRI)	NGS Results
4041-NP1139	31	F	Diffuse hypomyelination of the subcortical and deep white matter, enlarged high convexity CSF spaces, ponto-cerebellar hypoplasia, and thin corpus callosum.	Causative mutations
5078-NP1688	23	M	Slight peri- and supra-ventricular white matter hyperintensities and cortical atrophy.	Causative mutations
3277-CS130A	40	F	Diffuse hyperintensities in the corona radiata and centra semiovale.	Negative
3809-VR103A	26	F	Slight and diffuse signal alteration in the subcortical and deep white matter, and thin corpus callosum.	Negative
5303-VR813A	24	F	Large and irregular hyperintensities in the subcortical and perventricular with matter on T2-weighed and FLAIR sequences.	Negative

**Table 2:** Overview of genetic data.

Sample	Gene	Function	Genotype	Codon	Protein	Coding	HGMD ^a^	ACMG ^b^	Refs.
4041-NP1139	POLR3A	Missense	C/T	AGT	p.Gly1240Ser	c.3718G>A	Yes	L.P.	[10,11]
	POLR3A	Missense	G/T	AAG	p.Gln599Lys	c.1795C>A	Yes	L.P.	[10]
5078-NP1688	POLR3A	Missense	G/T	AAG	p.Gln599Lys	c.1795C>A	Yes	L.P.	[10]
	POLR3A	Missense	T/C	GAA	p.Lys110Glu	c.328A>G	-	L.P.	(c)

(a) HGMD Professional 2022.2, (b) ACMG Classification version 11.3.8, (c) this study. L.P.: Likely pathogenic.

**Table 3:** Predictive test of the c.328A > G (p.Lys110Glu) new mutation.

In Silico Predictive Tool	Prediction/Score/PHRED-Scaled
CADD_pred	27.2 (>30 highly pathogenic; >20 pathogenic)
DANN_score	0.999 (range from 0 to 1) *
FATHMM_pred	Tolerated
GERP++_RS	5.67 (range from −12.3 to 6.17) *
LRT_pred	Deleterious
M-CAP_pred	Damaging
MetaSVM_pred	Tolerated
MetaLR_pred	Tolerated
MutationAssessor_pred	Medium
MutationTaster_pred	Damaging
PROVEAN_pred	Deleterious
Polyphen2_HDIV_pred	Probably damaging
SIFT_pred	Deleterious
SiPhy_29way_logOdds	15.915 (range from 0 to 37.9718) *
Fathmm-MKL_coding_pred	Damaging
ACMG criteria	Likely Pathogenic **

* Higher value indicates a higher probability to be damaging. ** Analysis was performed with Varsome [8].

## Data Availability

Not applicable.

## Supplementary Materials

The following supporting information can be downloaded at: https://www.mdpi.com/article/10.3390/biomedicines10092276/s1, Supplementary Table S1: POLR3A mutations reported in HGMD database; Supplementary Table S2: Mutation and polymorphism results in-house NGS gene panel.
